# Identification of a Consolidation Phase in Immunological Memory

**DOI:** 10.3389/fimmu.2019.00508

**Published:** 2019-03-19

**Authors:** Francesca Mantile, Angelo Capasso, Piergiuseppe De Berardinis, Antonella Prisco

**Affiliations:** ^1^Institute of Genetics and Biophysics, CNR, Naples, Italy; ^2^Institute of Protein Biochemistry, CNR, Naples, Italy

**Keywords:** vaccine, boost, antibody, primary response, secondary response

## Abstract

Long lasting antibody responses and immunological memory are the desired outcomes of vaccination. In general, multiple vaccine doses result in enhanced immune responses, a notable exception being booster-induced hyporesponsiveness, which has been observed with polysaccharide and glycoconjugate vaccines. In this study, we analyzed the effect of early booster doses of multimeric protein vaccine (1-11)E2 on recall memory to B epitope 1-11 of β-amyloid. Mice immunized with a single dose of (1-11)E2 stochastically display, when immunized with a recall dose 9 months later, either memory, i.e., an enhanced response to epitope 1-11, or hyporesponsiveness, i.e., a reduced response. Memory is the most common outcome, achieved by 80% of mice. We observed that a booster dose of vaccine (1-11)E2 at day 15 significantly reduced the ratio between the magnitude of the secondary and primary response, causing an increase of hyporesponsive mice. This booster-dependent disruption of recall memory only occurred in a limited time window: a booster dose at day 21 had no significant effect on the ratio between the secondary and primary response magnitude. Thus, this study identifies a consolidation phase in immunological memory, that is a time window during which the formation of memory is vulnerable, and a disrupting stimulus reduces the probability that memory is achieved.

## Introduction

Vaccination affords immunity from diseases by inducing immunological memory and long-lived antibody responses (1, 2). The identification of switches that regulate immunity is central to efforts of rational vaccine design (3, 4).

Immunological memory, i.e., the ability to mount an enhanced response to an antigen that has been previously encountered, is a system-level property of the immune system, that arises from an increase in the frequency of antigen-specific B and T lymphocytes as well as from the differentiation of antigen-experienced lymphocytes into qualitatively different cell populations, namely memory cells, which display faster response to antigen re-exposure and the ability to self-renew (5–7). The half-life of the antibody titer, which is a critical issue in vaccine development as it is linked to the duration of protection, displays considerable variation among different vaccines currently in use. In humans, a longitudinal study of the antibody titer to common viral and vaccine antigens found that antibody responses against tetanus and diphtheria antigens waned more quickly, with estimated half-lives of 11 and 19 years, respectively, whereas antiviral antibody responses were remarkably stable, with estimated half-lives ranging from 50 years for varicella-zoster virus to more than 200 years for other viruses such as measles and mumps (8). The antibody titer in the circulation reflects the size of the antibody-secreting cells (ASC) pool, that includes different populations of ASC, that differ in their proliferative potential, life-span, and that are prominent in different temporal phases of the immune response, namely plasmablasts, short-lived plasma cells, and long-lived plasma cells (9). Of these, long-lived plasma cells ensure the long-term persistence of antibodies (10). Thus, the duration of antibody responses is related to the number and longevity of long-lived plasma cells. Survival vs. death of plasma cells is one of the key decisions that guide antibody production; understanding the control system of this decision, not only is potentially valuable for vaccine development, but also for treating disorders of antibody production in autoimmunity, allergy, and immunodeficiency (11).

The control system governing the quality and quantity of circulating antibody, far from being a single binary switch, comprises a series of decision points where B cells integrate many inputs influencing their fate (11); a crucial role is played by the Germinal Center (GC) reaction, a highly complex process involving a cascade of several distinct, timed events that are topographically segregated (12). The role of asymmetric cell division and stochastic events in this coordinated process of cellular differentiation and selection is still unresolved. Measures of the time to develop into a plasmablast, and to divide or die for thousands of cells suggested that each fate is pursued autonomously and stochastically and that the allocation of a proportion of B cell to each fate is a phenomenon of stochastic competition (13).

In this study, we set out to investigate the effect of the time delay between the first and the second dose of vaccine on the antibody titer trajectory during the primary and secondary response. Antibody titer/time curves reflect the contribution of antibody secreting cells that reside in different organs, namely the lymph nodes, the spleen, and the bone marrow, which become prominent in different time windows. While it is not feasible to analyze over time the development of different ASC populations in a single individual, serum can be sampled multiple times; thus, we took the approach of analyzing a single experimental parameter, namely the IgG antibody titer against a specific B epitope, in 50 genetically identical, age, and sex-matched mice over 1 year post vaccination. We monitored the primary response for 9 months, and then we administered a recall dose and monitored the secondary response for 3 months, sampling sera at 11 timepoints that we had previously identified as sufficient to capture the shape of the titer/time curve. We utilized as a model vaccine (1-11)E2, a multimeric protein designed to induce an antibody response against the β-amyloid peptide, a peptide involved in the pathogenesis of Alzheimer's Disease (14, 15). (1-11)E2 is an icosahedral protein nanoparticle, displaying 60 copies of peptide 1-11 of β-amyloid, at the N-terminus of self-assembling protein domain E2 (16). A single injection of (1-11)E2 induces recall memory to the displayed β-amyloid epitope in the majority of immunized subjects (16), making this multimeric protein a suitable antigen for the investigation of recall memory.

Mice immunized with a single dose of (1-11)E2 stochastically display, when immunized with a recall dose 9 months later, either memory, i.e., an enhanced response to epitope 1-11, or hyporesponsiveness, i.e., a reduced response. Memory is the most common outcome, achieved by 80% of mice.

When a booster dose of vaccine (1-11)E2 was administered at day 15, we observed a significant reduction of the ratio between the magnitude of the secondary and primary response, resulting in an increase of hyporesponsive mice. This booster-dependent disruption of recall memory only occurred in a limited time window: a booster dose at day 21 had no effect on the ratio between the secondary and primary response magnitude.

Hyporesponsiveness, defined as a lower antibody (Ab) level after the second immunization than after the first, has been observed after vaccination with polysaccharide or glycoconjugate vaccines (17). We report here, for the first time in our knowledge, that hyporesponsiveness also occurs in the case of a multimeric protein antigen and can be induced by a booster dose administered in a specific time window.

Thus, this study identifies a consolidation phase in immunological memory, that is a time window during which the formation of memory is vulnerable, and a disrupting stimulus reduces the probability that memory is achieved.

## Results

### A Booster Dose Given 15 Days After Priming Impairs Immunological Memory to a B Cell Epitope

In this study, we set out to investigate the effect of the timing of a booster dose on immunological memory to a B cell epitope. Our model epitope is Aβ(1-11), consisting of the 11 amino acid N-terminal immunodominant B epitope of β-amyloid. Immunization against Aβ(1-11) was performed with antigen (1-11)E2, a recombinant protein comprising epitope 1-11 of β-amyloid and the E2 domain of the pyruvate dehydrogenase of *Bacillus stearothermophilus*, that self-assembles into a multimeric structure that includes 60 monomers (14).

For this study, we define memory as the ability to display an enhanced response to an antigen that has been previously encountered. In particular, in this study, the feature of the immune response that we analyze is the IgG antibody titer.

**Figure 1:**
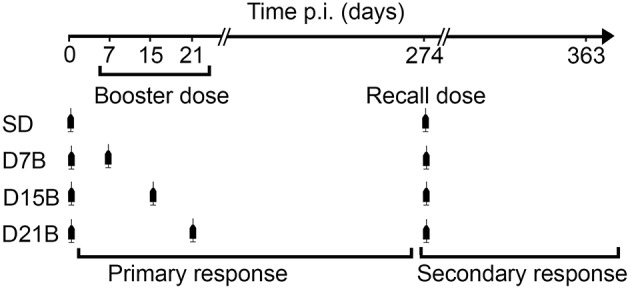
Experimental design. 50 BalbC mice were randomly allocated to four immunization schedules. The control group received only a single dose (SD) of vaccine (1-11)E2, while treatment groups D7B, D15B, and D21B also received a booster dose, respectively, at day 7, 15, or 21 after the first dose. All mice received a recall dose 9 months after the first dose and were then monitored for 3 more months. The IgG antibody titer against Aβ(1-11) was monitored for 1 year post-immunization. The definition of primary and secondary response is shown: the primary response is defined as the response initiated by the first dose of vaccine (days 0–274 post immunization), the secondary response is defined as the response initiated by the recall dose (days 274–363 post immunization).

We monitored for 1 year post-immunization the IgG antibody titer against Aβ(1-11), in 50 mice undergoing a primary and a secondary response. The experimental setup and the definition of primary and secondary response are schematized in Figure 1. Mice were randomly allocated to four immunization schedules. The control group received only a single dose (SD), while treatment groups D7B, D15B, and D21B also received a booster dose, respectively, at day 7, 15, or 21 after the first dose. All mice received a recall dose 9 months after the first dose and were then monitored for 3 more months (Figure 1).

**Figure 2:**
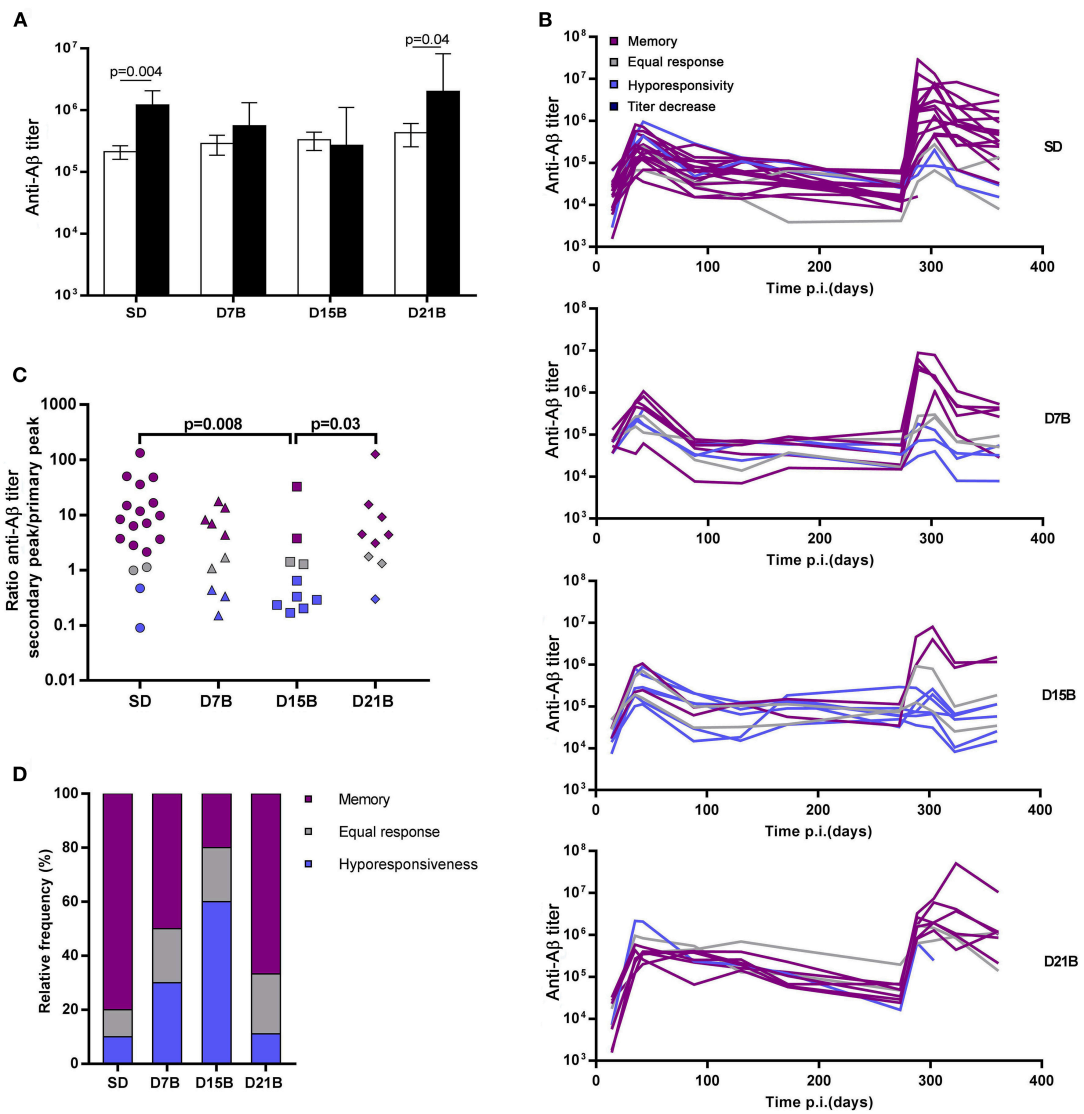
Effect of booster doses on recall memory. **(A)** The histograms show the geometric mean titer of IgG against Aβ(1-11) at the peak of the primary response (open bars) and secondary response (black bars). Error bars represent the standard error of the mean (s.e.m.). Significant *P*-values calculated with the Wilcoxon rank sum test are shown. **(B)** The line graph shows the time course of the IgG titer against Aβ(1-11) in individual mice. Trajectories are color-coded based on the classification of response patterns as in **(B,C)**. **(C)** The dot plot shows the ratio between the peak titer of IgG against Aβ(1-11) in the secondary response and the primary response in individual mice. Each symbol represents one mouse. Significant *P*-values calculated with the Wilcoxon rank sum test are shown. **(D)** The histogram shows the relative frequencies of 3 patterns of response to recall, defined based on the ratio of the peak of the secondary response to the peak of the primary response as memory (ratio > 2, violet), equal response (0.5 ≤ ratio ≤ 2, grey) hyporesponsiveness (ratio < 0.5, blue).

In order to establish whether the different treatment groups had developed immunological memory to Aβ(1-11), defined as the ability to display an enhanced recall response, we compared, within each group, the magnitude of the peak of the primary and secondary antibody response to Aβ(1-11) (Figure 2A). While the mice that received a single dose of vaccine and the group that received a booster dose at day 21 displayed a significantly enhanced peak response to the recall dose, we observed no statistically significant difference in magnitude between the peaks of the primary and secondary total IgG response in the groups that received the booster dose at day 7 or 15. Thus, a booster dose administered within 15 days of the first dose abrogated immunological memory, defined as the ability to display an enhanced recall response.

Indeed, the effect of the booster dose given at day 7 on recall memory appeared less severe than the effect of the booster dose given at day 15. As shown in Figure 2A, both in the D7B and in the D15B group there is no statistically significant difference between the secondary and primary response peak, however the geometric mean titer of the secondary response displays a trend toward higher values in the D7B group (Figure 2A).

Among mice of the same treatment group, we observed a broad spread of the anti-Aβ(1-11) IgG titers at the peak of the secondary response (Figure 2B). In order to analyze the diversity of the fate of the immune response between individuals, we classified individual titer/time trajectories with respect to the ratio between the peak of the secondary response and the peak of the primary response, so as to be able to recognize “immunological memory,” defined as an enhanced secondary response, at the level of the individual.

The ratio between the peak of the secondary response and the peak of the primary response ranged from 0.1 to over 100 (Figure 2C). We defined “memory” a secondary response 2-fold higher than the primary response, that is a ratio of the antibody titer of the secondary peak to the primary peak above 2, “equal response” a ratio comprised between 2 and 0.5, and “hyporesponsiveness” a ratio lower than 0.5.

All treatment groups included some mice that had developed memory to Aβ(1-11), albeit at different frequencies (Figures 2C,D). The ratio between the secondary and the primary peak was significantly lower in the D15B group, compared to the SD group and the D21B group (Figure 2C). The number of mice that displayed a memory response to Aβ(1-11) was minimal in the D15B group (Figure 2D).

In the analysis of the ratio between the secondary peak and the primary peak in individual mice (Figure 2C), while the D15B group is statistically different from the SD group (*p* = 0.008), the difference between the D7B group and the SD group is not statistically significant. In the classification of individual recall responses shown in Figure 2D, the D7B group appears intermediate between the SD group and the D15B; in the D7B group the percentages of mice displaying memory was lower than in the single dose group, but higher than in the D15B group (Figure 2D), whereas conversely in the D7B group the percentage of mice displaying hyporesponsiveness was higher than in the SD group but lower that in the D15B group.

In summary, only when the booster dose is given at day 15 there is a statistically significant reduction in the ratio between the magnitude of the secondary and primary response.

### Hyporesponsiveness Is Unrelated to the Primary Response and to Antibody Titer at Recall

**Figure 3:**
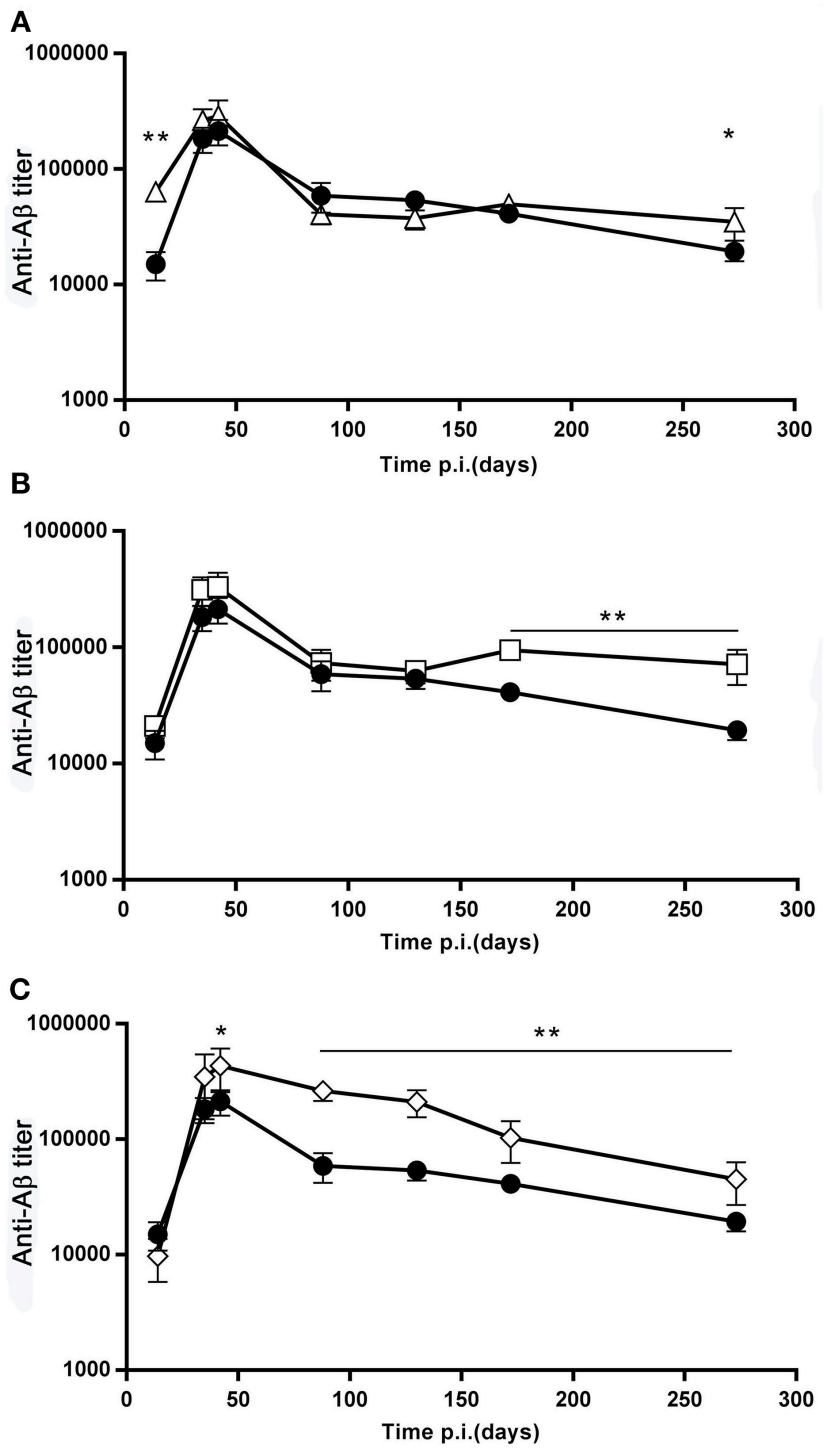
Effect of booster doses on the primary response. **(A–C)** The graphs report the time course of the anti β-amyloid antibody titer, GMT ± SEM, of the SD group (full circles, *N* = 20), overlayed to the time course of the D7B group (open triangles, *N* = 10, **(A)**, the D15B group (open squares, *N* = 10, **(B)**, and the D21B group (open diamonds, *N* = 10, **(C)**. ^*^p ≤ 0.05, ^**^p ≤ 0.02.

The antibody titers from day 0 to 274, shown in Figure 3, demonstrate that, differently from the recall response, the primary response was not reduced in the mice that received booster doses, compared to the mice that received only a single dose (Figure 3).

Moreover, we asked whether the ability to exhibit an enhanced response to the recall dose was related to the antibody titer at the time of recall.

**Figure 4:**
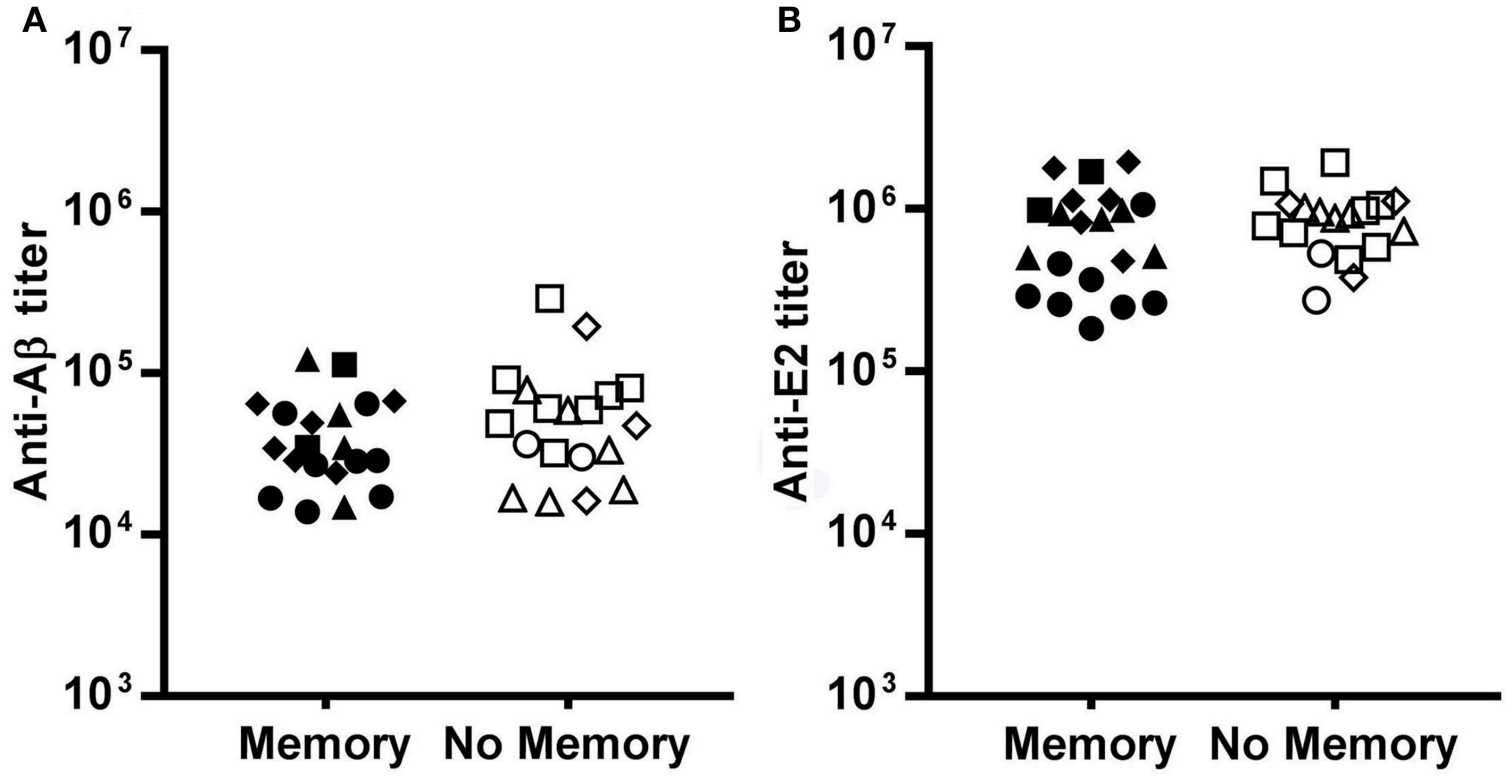
Pre-existing serum titers at recall. The dot plots show the IgG titer against Aβ **(A)** and E2 **(B)** at day 273, the day before the recall dose, in mice that displayed memory or no memory against Aβ. Each dot represents a mouse of the SD group (circles), D7B group (triangles), D15B group (squares), D21B group (diamonds). There is no statistically significant difference between memory and no memory mice as regards the antibody titer against Aβ and E2.

We observed no significant difference in the anti-Aβ(1-11) antibody titer at the time of recall between mice that displayed a memory response to Aβ(1-11) and mice that did not (Figure 4A). Mice immunized with (1-11)E2 develop an antibody response both to the Aβ(1-11) peptide and to the scaffold protein domain E2. The antibody titer against E2 at the time of recall also did not differ between mice with and without memory to Aβ(1-11) (Figure 4B). Thus, the different fates in individual responses to the recall dose were not related to differences in the titer of circulating antibodies against the immunizing antigen at the time of recall.

### Recall Memory to the E2 Carrier Protein Is Impaired by a Day 15 Booster

**Figure 5:**
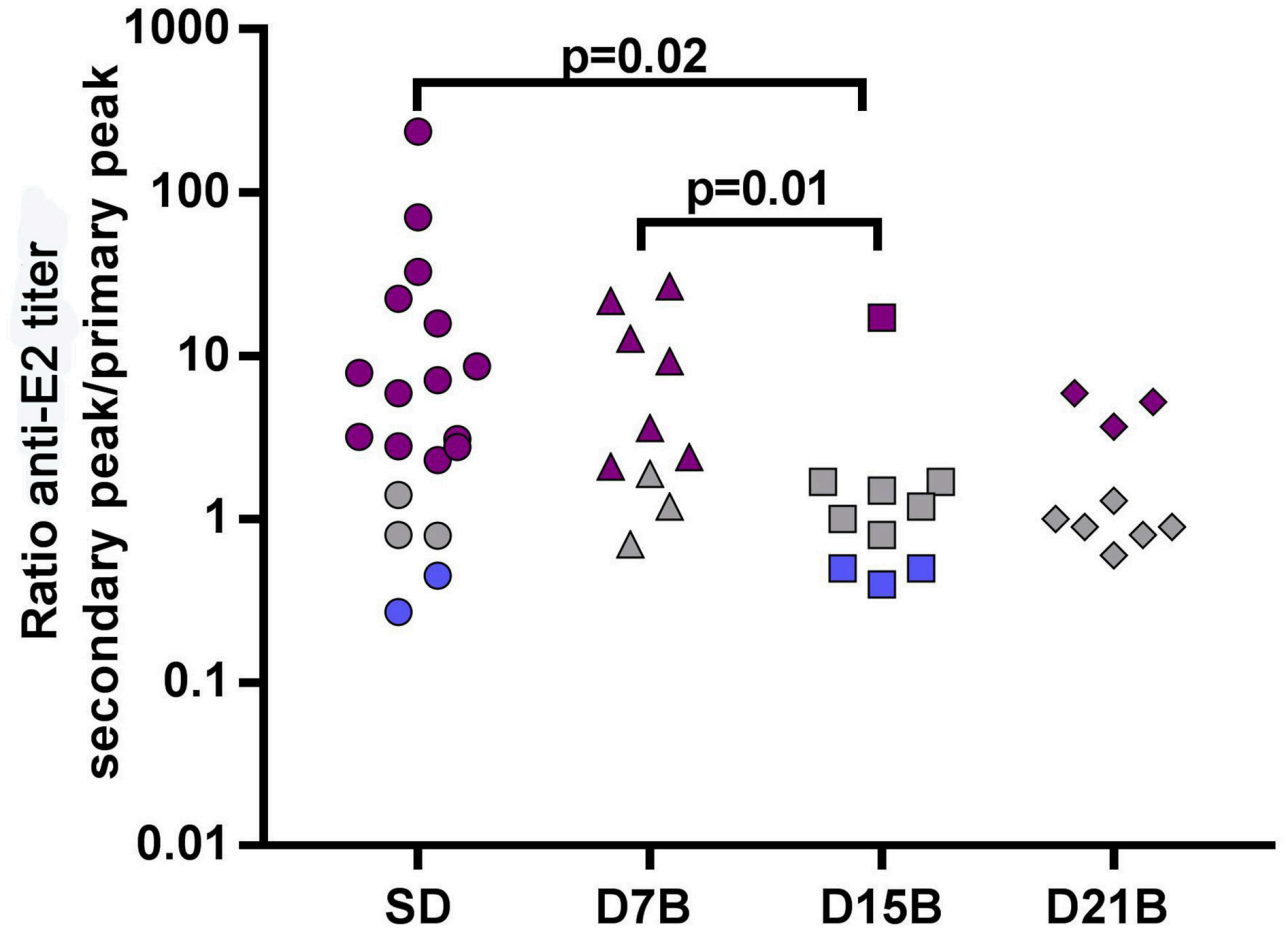
Effect of booster doses on recall memory to E2. The dot plot shows the ratio between the peak titer of IgG against E2 in the secondary response and the primary response in individual mice. Each symbol represents one mouse. The same color code of Figure 2 is used: violet (memory), grey (equal response), blue (hyporesponsiveness). Significant *P*-values calculated with the Wilcoxon rank sum test are shown.

We asked if the booster-related effects on recall memory were limited to the Aβ(1-11) B cell epitope or extended to other B epitopes of the immunizing antigen (1-11)E2. Thus, we analyzed the IgG antibody titer trajectories against the carrier moiety E2. In accordance to what we observed in the response to the β-amyloid epitope (1-11), also in the response to the E2 protein the ratio between the peak of the secondary and primary response is significantly reduced (*p* = 0.02) in the group that received a booster dose at day 15, compared to the single dose group (Figure 5).

Both in the single dose group and in the group that received the day 15 booster, the ratio between the secondary and primary response peak is highly correlated between the response to Aβ(1-11) and the response to E2 (Pearson correlation coefficient is 0.94 in the single dose group, and 0.99 in the D15B group).

This results demonstrate that the day 15 booster dose impaired recall memory not only to the Aβ(1-11) epitope, but also to other B epitopes of the E2 carrier.

## Discussion

The most notable finding in this study is that a booster dose of the multimeric protein antigen (1-11)E2, injected 15 days after the primary immunization, impaired the antibody response to a recall dose, administered 9 months later. In particular, the analysis of the trajectories of the antibody titer against B epitope Aβ(1-11) in individual mice revealed that a booster dose at day 15 resulted in fewer mice being subsequently able to exhibit an anamnestic response to the recall dose, and in some mice displaying hyporesponsiveness. While in the single dose group only 4/20 mice failed to mount an enhanced secondary response, in the day 15-boost group this happened in 8/10 mice. On the other hand, a booster dose given at day 21 after the primary immunization did not affect the fold ratio between the secondary and primary response.

It is possible to speculate that in our experiment the booster dose interfered with a different stage of the GC reaction, depending on its precise timing. Pre-existing GC can be populated by new B cell clones following a booster immunization (18, 19). It has been suggested that B cells that acquire antigen can enter GCs at all stages of the response, and that antigen is one of the main limiting factors (18). The GC response undergoes a temporal switch in its output; memory B cells and long-lived plasma cells are produced at separate points in time (20). In particular, unswitched memory B cells are generated early in the response, followed by switched memory B cells, and finally by a delayed appearance of isotype-switched bone marrow long-lived plasma cells (20). We never observed, in prime-boosted mice, a reduced primary response compared to single dose mice, indicating that booster doses did not inhibit ASC development, as shown in Figure 3.

Neutralizing serum immunoglobulin can inhibit the secondary response and have differential effects on B cell populations that mediate early and late memory (21). In our experiment the antibody titers at the time of the recall dose were in the same range in mice that then demonstrated an enhanced secondary response (memory) and in those that did not; therefore we can rule out that circulating antibodies inhibited the secondary response.

From our experiment, it is not possible to establish whether an impaired development of memory cells or a dominant inhibitory mechanism caused the observed hyporesponsiveness.

Several studies have reported that booster doses of polysaccharide vaccines can induce unresponsiveness. Unconjugated meningococcal polysaccharide vaccination induces antibody hyporesponsiveness, that impairs antibody responses to subsequent injections of meningococcal polysaccharide (MPS) or meningococcal conjugate vaccines. Administering MPS as a probe to assess conjugate vaccine-induced immunologic memory also can extinguish subsequent memory anticapsular antibody responses, whereas conjugate vaccination regenerates memory B cells (22). A mechanism that has been proposed for the hyporesponsiveness caused by polysaccharide antigens is that the polysaccharide, a T independent antigen, may stimulate the existing pool of memory B cells to differentiate into plasma cells and secrete antibody without replenishment of the memory B cell pool (22). A study on the effect of 1, 2, or 3 boosters of pneumococcal polysaccharide with 16 day intervals, in mice primed with a pneumococcal conjugate concluded that booster-induced hyporesponsiveness is caused by abrogation of conjugate-induced GC reaction and depletion of polysaccharide-specific Antibody-secreting cells, resulting in no homing of new specific long-lived plasma cells to the bone marrow (23). At difference with our study, the pneumococcal polysaccharide booster reduced the antibody titer of boosted mice, compared to the PBS control; instead, we did not observe a titer reduction. A difference in the study design is the age of the mice at the time of priming. The study on the effect of pneumococcal polysaccharide was performed on neonatal, 7 days old mice, whereas our study was performed on adult, 8 weeks old mice.

For human vaccines currently in use, the minimum interval to next dose recommended by the Advisory Committee on Immunization Practices is between 4 weeks and 5 years. The day 15 boost has been widely utilized to vaccinate mice against β-amyloid with β-amyloid 1–42 (24) recombinant bacteriophages (25) and recombinant proteins (14). Agent-based simulations of the response to our model vaccine predicted that a booster dose would be inefficient if given earlier that a few months after the first dose (26), however, the study did not investigate booster-induced unresponsiveness to recall.

The results of this study show that there is a consolidation phase in immunological memory to the Aβ(1-11) epitope; there is a time window, after immunization with the vaccine (1-11)E2, during which the fate of the secondary response to the Aβ(1-11) epitope is vulnerable, and a disrupting stimulus reduces the probability that memory is achieved.

Interestingly, the results we obtained analyzing the antibody response to the β-amyloid epitope and the carrier epitopes were similar, in that a booster injection at day 15 caused a reduced probability of a subsequent enhanced secondary response to both the β-amyloid and the E2 carrier protein. In our classification of responses as memory, equal response and hyporesponsiveness, some mice fall into a different as regards the response to (1-11) and the response to E2. A possible explanation for this discordance lies in the fact that the E2 response reflects the cumulative behavior of more cells, and therefore more often falls into the intermediate pattern, i.e., “equal response.” In fact, E2 is a larger antigen than Aβ(1-11), comprising 257 amino acids vs. 11 amino acids, and the response to E2 reaches a titer 7 times higher than the response to Aβ(1-11), indicating that more clones are involved in the response to E2 than in the response to Aβ(1-11).

A word of caution is needed regarding the generalization of the kinetics that we observed, as it is possible that different types of antigen, adjuvants, or injection routes, and different dose may be associated with differences in the kinetics of the response.

This study paves the way to investigating early correlates of immunological memory development, by analyzing the molecular and cellular effects of memory-disrupting stimuli.

## Materials and Methods

### Mice

All experiments were performed on female BalbC mice. Mice were purchased from Charles River Laboratory, Italy. The first dose of vaccine was injected when the mice were 8 weeks old.

### Model Vaccine

The vaccine (1-11)E2 is a multimeric protein. The monomer, that self-assembles into a 60-mer complex, consists of a fusion protein that includes the first 11 N-terminal residues of the β-amyloid peptide, DAEFRHDSGYE, and a bacterial protein domain, from the E2 subunit of the Acyl-transferase of *Bacillus stearothermophilus* (14, 15). The (1-11)E2 protein was produced in E. coli and purified and stored as previously described (14, 15).

Each vaccine dose consisted of 130 μg of (1-11)E2 protein (carrying 6 μg of the β-amyloid epitope 1-11) mixed with 100 μl of Freund's adjuvant, in a final volume of 200 μl. Complete Freund's adjuvant was used in the first injection, and incomplete Freund's adjuvant was used in subsequent shots. The vaccine was injected intraperitoneally.

### Immunization and Bleeding Schedules

We have monitored, for a total of 12 months, the time course of the antibody response in 50 individual BalbC mice, undergoing 4 different dosing schedules. All dosing schedules included a first dose given when the mice were 2 months old, and a recall dose given 9 months after the first dose. Twenty mice only received these 2 doses, while other groups, of 10 mice each, also received a booster dose, respectively, 1, 2, or 3 weeks after the first dose.

Blood was collected from the tip of the tail, with heparinized microhematocrit capillaries, at the following time points after the first dose: day 14, 35, 42, 88, 130, 172, 273, 288, 302, 323, and 361. Blood was left at room temperature for 30 min, then centrifugated at 6,000 rpm for 30 min. The serum was divided into aliquots and stored at −80°C.

### Antibody Titer Measures

The antibody titer was measured by ELISA assays, performed as previously described (15).

Each serum was tested against synthetic peptide 1-11 of β-amyloid. Synthetic peptide 23–29 of β-amyloid was used as a negative control. The titer of serum was defined as the dilution yielding an absorbance value equal to 2-fold the background value obtained against the negative control.

### Statistical Analysis

The Wilcoxon rank sum test was performed to determine the statistical significance of observed differences.

## Ethics Statement

This study was carried out in accordance with European Union Laws and guidelines (European Directive 2010/63/EU) and in accordance with the authorization 161/2015-PR released by the Italian Ministry of Health.

## Author Contributions

AP contributed to the conception and design of the study and wrote the manuscript. FM and AC performed the experiments. FM, AC, PDB and AP contributed to the data analysis, interpretation, and manuscript revision, and read and approved the submitted version.

### Conflict of Interest Statement

The authors declare that the research was conducted in the absence of any commercial or financial relationships that could be construed as a potential conflict of interest.
